# Doctors in Fiction

**Published:** 1887-07-09

**Authors:** C. J. Furley


### Doctors in Fiction.

By C. J. Furley.

As a rule medical men gain but scant attention from novelists; they come in at crises of the tale as beings little superior to the *personæ mutæ* of the stage, pronounce their verdict of bliss or bale, and then depart into space. A popular lady novelist of our time occasionally draws a fancy picture of the village surgeon smoking his pipe in the village tavern, and gossiping in a very unprofessional way of the ailments and fortunes of the squire and his family; but even this mythical personage plays but a subordinate part in the evolution of the story: indeed, does little more than act as a sort of chorus, provided, in Wagnerian fashion, with an ever-recurring *leit motif* of "*digitalis*," "*cum grano salis*," and a few more vaguely therapeutic phrases promiscuously applied.

The other professions receive more consideration from the average story-teller. Not to speak of the ever-recurring brown-bearded, picturesque young artist; and the dreamy, mystically inspired poet, who at twenty-five has written a drama or epic (discreetly left unquoted) which has thrilled society to the core; the curate of domestic romance, and the lawyer of melodrama, are all made much more of than the elderly—always elderly—"medicine man," grave, solemn, and "professional in manner" (whatever that may mean), who declares that the heroine will recover from her death-like swoon, or that the aged miser can grasp his sin-stained money-bags for but a few hours longer.

Perhaps there is a wisdom in this. The literary practice of medicine bristles with dangers for the unwary; for while the pictures may be described vaguely and safely as "Titianesque in colouring," or "worthy of Raphael in his most inspired moments," and the poetry may be left to the reader's imagination; while nobody asks if the curate's theology is orthodox, or the lawyer's opinion sound, so long as they help to bring the tale to a happy conclusion, there is a tendency to deal harshly with the novelist who too recklessly kills his villain by fracture of the radius, or cures his hero of tubercular meningitis. Of course one must not exact too great accuracy in the treatment of symptoms, and it must be permitted to heroes and heroines to survive accidents which would kill ordinary folks, or where would your third volume be? I cannot say that I sympathise with the extremists, who will not enjoy an otherwise interesting tale because of a single inaccuracy in medical fact. A conscientious oculist told me that he could not read Hugh Conway's "Called Back," because the author, after minutely describing the operation for cataract, rashly avers that in time the hero will be able to do without very thick and ugly spectacles.

"It is utter nonsense!" he exclaimed, indignantly. "The author talks of training the eye to do its old work without artificial aid. We aren't spiders, to manufacture new limbs at will, and no training will teach the eye to create a new crystalline lens in place of the one which has become opaque, and which the surgeon has removed. The ugly goggles are the only possible substitute."

This gentleman was an extremist, but one of a pardonable sort. The unpardonable type is represented by that otherwise estimable physician—I must confess that he hails from over Tweed—who sent me back Louis Stevenson's wonderful romance, "Dr. Jekyll and Mr. Hyde," with the remark that the transformation on which the story is based was "quite impossible."

There are indeed several incidents of fictitious medicine (if one may dare so to entitle it) which we would all like to believe: for example, that a gentleman may be shot through the lungs and yet be able to lead a siege party a month afterwards, inspiring the wavering ranks with courage and confidence by the sound of his clarion voice. Also we would gladly credit the statement that "Sir Guy Bartholomew," or any other ingeniously-named consultant, gets a five-hundred-guinea fee every time he is called from London to—say Richmond. If this were the case it would be exceedingly good news for the medical profession in general, and especially for Sir Guy Bartholomew. Doubtless the writer is following medical advice in such generosity. Wendell Holmes recommends that in depicting a fictitious character "you should give him all you can, it costs you nothing."

Another characteristic of the story-teller in dealing with doctors and their work is his reckless and interchangeable use of the titles physician and surgeon. As a rule, we find that a surgeon is considered quite able to cure a common scarlet fever or vulgar whooping-cough; but if a limb has to be amputated, a physician must needs be called in. These distinctions tend to enlighten the professional reader with regard to lay comprehension of his qualifications. According to the novelist, too, typhoid fever is a highly infectious disease; and superfluous personages habitually die of heart-disease at convenient moments, usually with a dramatically unfinished speech upon their lips. I do not think there has ever been any attempt made to tabulate the modes of death in fiction; but I feel convinced that at least ninety per cent. of the characters are killed by heart-disease.

The danger of falling into errors more obvious than these doubtless keeps many novelists from attempting full-length portraits of medical men, it being so difficult to portray the man apart from his work; but our literature can supply us with at least a dozen types of doctors, clearly drawn, both the good and the bad.

Let Doctor Sangrado, of whom Gil Blas tells us, be accorded the first place on the list, for he represents an extinct species. His name tells us his most marked characteristic. Unlike "Hamlet's aunt," whose conversation so startled David Copperfield, he did not believe in blood—at least so long as it pulsed through human veins. "It is a mistake to think that blood is necessary to the preservation of life," he said; "one cannot bleed a patient too much." Accordingly he practises phlebotomy on the poor canon who has entrusted his life to his care, till there is scarcely a drop of blood left in the patient's body. The canon dies; but there is no record of the doctor renouncing his favourite theory because of the accident. Theories are precious, even when their results in practice are unfortunate; and Dr. Sangrado and those who shared his sanguinary creed died hard.

(*To be continued.*)

